# Endovascular Aneurysm Repair Complicated with Type Ia Endoleak and Presumable Infection Treated with a Fenestrated Endograft

**DOI:** 10.1055/s-0039-1681067

**Published:** 2019-03-08

**Authors:** Arne de Niet, Paul M. van Schaik, Ben R. Saleem, Clark J. Zeebregts, Ignace F. J. Tielliu

**Affiliations:** 1 Division of Vascular Surgery, Department of Surgery, University of Groningen, University Medical Center Groningen, Groningen, The Netherlands

**Keywords:** popliteal aneurysm, aortic aneurysm, *Listeria monocytogenes* infection, fenestrated endovascular aneurysm repair

## Abstract

An 81-year-old patient presented to the emergency room 5 years after infrarenal endovascular aneurysm repair, with a Type Ia endoleak and a presumable infection of the graft material with *Listeria monocytogenes* . He was treated with a custom-made fenestrated endograft to seal the endoleak and lifelong antibiotic therapy to suppress the infection. Full explantation of graft material is not always preferable, and endovascular treatment combined with antibiotic suppressive therapy is in some cases an appropriate alternative.

## Introduction

Treatment of an abdominal aortic aneurysm (AAA) by endovascular aneurysm repair (EVAR) has good postoperative outcome. 1 Long-term risks include migration of the endograft and Type Ia endoleak, whose occurrence depends on anatomic characteristics of the aortic neck and the type of endovascular device used. 2 3 Infection of the implanted endograft can also occur. 4 There is general agreement that invasive treatment is the best option to treat these infections because of an increased risk of rupture compared with when they are left untreated. 5 The most durable solution includes full explantation of the endograft. This is not always feasible because of patient characteristics, including aortic anatomy, hostile abdomen, and comorbidities. As a consequence, long-term antibiotic suppressive therapy may serve as the second best.

We present a case of a Type Ia endoleak after EVAR in a patient highly suspected of having an infection of the endograft.

## Case Presentation

An 81-year-old man presented to the emergency room with fever, 5 years after EVAR for an infrarenal AAA of 54 mm in diameter with a Gore Excluder AAA Endoprosthesis (W.L. Gore & Associates, Inc., Flagstaff, AZ). During follow-up, a Type II endoleak in a stable aneurysm sac diameter was accepted. Three years prior to presentation, the patient was treated with an infragenicular femoropopliteal polytetrafluoroethylene (PTFE) bypass for an acute occlusion of a left popliteal artery aneurysm (PAA). A PAA on the right side was treated with a reversed autologous saphenous vein bypass. Prior to presentation, the patient was treated with ciprofloxacin by his family physician for a urinary tract infection. In-hospital urine cultures taken at presentation showed ciprofloxacin-resistant infection with *Klebsiella pneumoniae* .

**Fig. 1:**
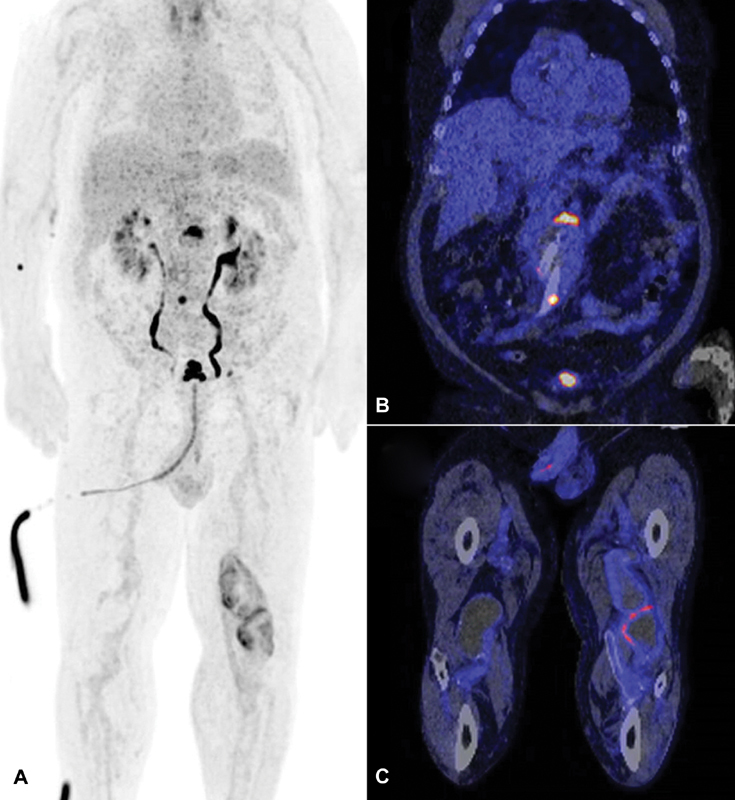
Coronal orientation of ^18^ F-fluorodeoxyglucose ( ^18^ F-FDG) positron emission tomography (PET) ( **A** ) and CT scan ( **B** and **C** ). Clear uptake in the ^18^ F-FDG–PET scan can be seen at the upper part (maximum standard uptake value [SUV _max_ ] 10.74; tissue-to-background ratio [TBR] 7.83) and at the bifurcation (SUV _max_ 8.28; TBR 6.04) of the endoprosthesis, and at the level of the treated left popliteal aneurysm (SUV _max_ 6.54; TBR 4.77).

At presentation the patient had fever up to 40°C. Laboratory examination showed plasma C-reactive protein (CRP) level of 254 mg/L and white blood cell count (WBC) of 12.3 × 10 ^9^ /L. Because of an unclear focus for the infection, ^18^ F-fluorodeoxyglucose–positron emission tomography ( ^18^ F-FDG–PET), combined with computed tomography angiography (CTA), was made. Increased FDG uptake on the PET scan was seen at the level of the proximal end and bifurcation of the aortic endograft and at the left PAA sac. All three locations had clear elevations in maximum standard uptake value (SUV _max_ ) and tissue-to-background ratio ( Fig. 1 ). In addition, a Type Ia endoleak was diagnosed with an increase in the aneurysmal sac diameter from 66 to 72 mm as compared with the duplex ultrasound 14 months earlier.

**Fig. 2:**
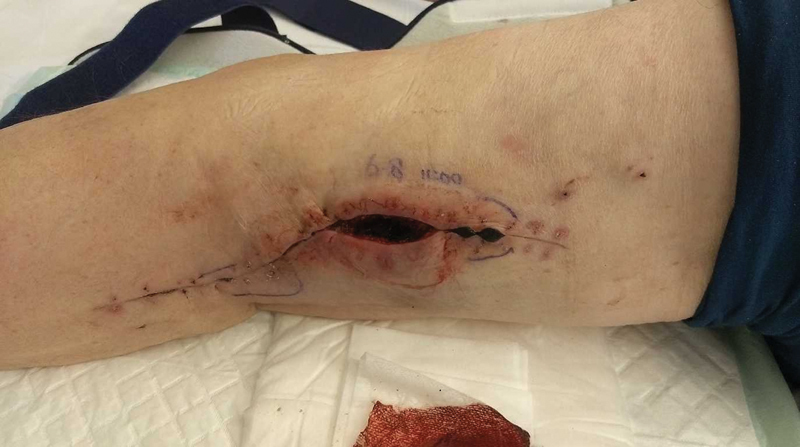
Photograph after opening and cleaning the infected left popliteal aneurysm. Delayed wound healing can be seen and central wound separation after staple removal. Secondary wound healing was later seen at the outpatient clinic.

Oral ciprofloxacin was switched to intravenous meropenem for wider pathogen coverage at admission. Puncture of the left PAA was done 8 days after admission. Culture of the aspirated material revealed *Listeria monocytogenes* only intermediately sensitive to meropenem, but sensitive for amoxicillin and cotrimoxazole, after which the antibiotic regimen was changed to this combination. After this switch, the fever subsided. The left PAA sac was opened surgically and cleaned ( Fig. 2 ). Amoxicillin was administered for 4 weeks intravenously and oral cotrimoxazole lifelong. Ten weeks after presentation, fever was still absent, plasma CRP level declined to a level of 38 mg/L, and WBC declined to 5.2 × 10 ^9^ /L.

**Fig. 3:**
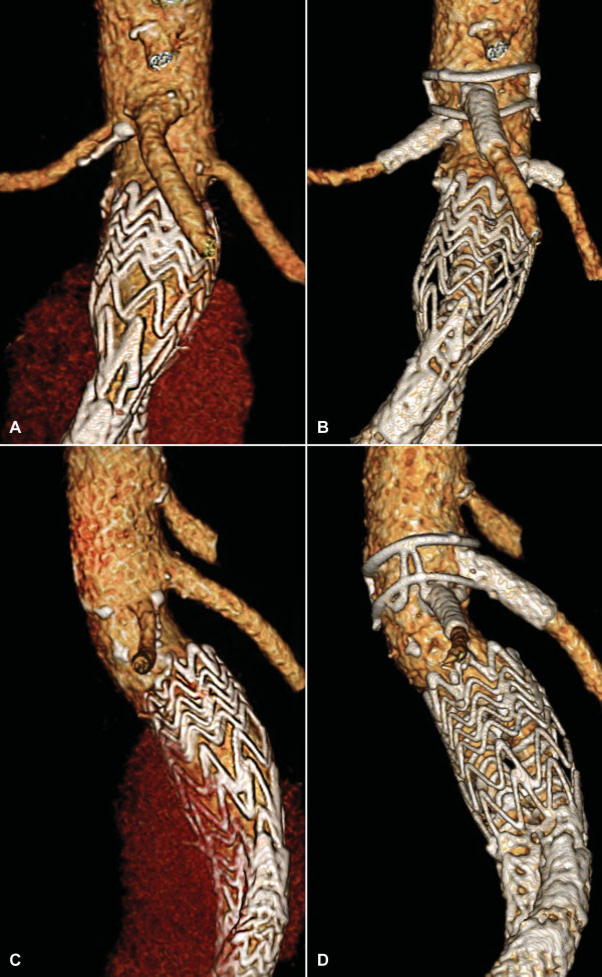
Before (left) and after (right) placement of the fenestrated Anaconda abdominal aortic aneurysm stent graft. Before treatment, another endograft for infrarenal endovascular aneurysm repair and an endoleak can be seen ( **A** , **C** ). After placement of the bi-iliac fenestrated Anaconda with stents inside the both renal arteries and the superior mesenteric artery, no endoleak was present ( **B** , **D** ).

Considering the patient's age and infections of both abdominal and popliteal vascular graft, an open surgical procedure to treat the Type Ia endoleak and remove the endograft was not an option. The infrarenal sealing zone was only 8 mm in length; therefore, repair with a fenestrated Anaconda AAA stent graft (Vascutek Ltd. Inchinnan, Scotland, United Kingdom) was chosen. The bi-iliac device contained fenestrations for both renal arteries and a fenestration for the superior mesenteric artery situated between the two proximal sealing rings ( Fig. 3 ).

For this procedure, a cutdown of both groins was performed to get access to both common femoral arteries. All three fenestrations were stented successfully, and no endoleak was present at completion angiography. The postoperative period was uneventful, and the patient was discharged from the hospital 3 days later. He is kept under lifelong oral antibiotic therapy with cotrimoxazole. CTA at 6 weeks did not show any endoleak or graft failure. After 3 months, no fever or events were reported, plasma CRP level was 9 mg/L, and WBC 5.3 × 10 ^9^ /L.

## Discussion

This case demonstrates the difficulties that may occur in clinical decision making when patients unfit for open repair are faced with multiple problems such as a large Type Ia endoleak after EVAR presumably complicated by an infection of the endograft.

Endovascular graft infection is rare, occurring in 0.6% of the cases and fatal in 63% when left untreated. 4 The greatest risk is contamination during the primary surgical procedure. Most infectious organisms are similar to those cultured in mycotic aneurysms, being *Staphylococcus aureus* in 43%, *Escherichia coli* in 17%, and *Staphylococcus epidermidis* in 10% of the cases. 4 6

Physical examination, chest X-ray, urine analysis, and blood cultures are standard to search for the focus in patients with suspicion of an infection. When these patients have concurrent endovascular grafts, colonization should be suspected, if the focus cannot be found or they are unresponsive to treatment. Standard follow-up of patients with an endovascular graft is usually done with CTA and duplex ultrasound, but these modalities do not always accurately show endovascular graft infections. The use of ^18^ F-FDG–PET scanning, combined with CT scanning, can help diagnose endovascular graft infections. 7

An infection with *L. monocytogenes* is also uncommon, and presentation can vary from fever and gastroenteritis to sepsis and meningitis. The origin is usually food based. Moreover, cattle farm residents have a higher risk of infection. 8

An endograft infected with *L. monocytogenes* has only been described in two cases, emphasizing the unusual combination. In the first case, it concerned a 77-year-old patient, treated by en bloc resection of endograft and aneurysm and subsequent reconstruction. 9 The second case described by our group was a 67-year-old man in whom fluid collections around the endograft, based on a *L. monocytogenes* infection, were successfully drained and treated with oral antibiotic therapy. 10 After more than 8 years follow-up, the patient died of lung carcinoma.

After EVAR, migration of the endograft and Type Ia endoleak have been shown to occur in up to 8.6% and 12.3% of the cases, respectively. 2 3 Treatment of these complications may also be performed by an endovascular approach, but a short infrarenal neck or even proximal extension beyond the renal arteries limits the possibilities. The use of a custom-made fenestrated endograft has good results, preventing the need for open surgery. 11

Treatment of the infected endograft would ideally mean removal of the infected foreign bodies, but it carries considerable mortality and morbidity risks. 12 Not in all cases, especially in endovascular prostheses, removal of infected material is possible and sole treatment with antibiotics is an option, especially with low virulent causative microorganisms. 13 The additional challenge in this case was a Type Ia endoleak, requiring treatment to prevent rupture. Placement of a fenestrated endograft in an infected area would imply that the newly implanted device also becomes infected. Cleansing accessible areas reduces the chance of bacterial overgrowth. By treating the patient with a maintenance dose of antibiotics, the infectious agent can be controlled, but bacterial resistance can be a risk. The patient should be monitored closely for device failure and relapse of infection despite suppressive therapy.

Fenestrated EVAR for an infected infrarenal endograft and concomitant Type Ia endoleak is technically feasible. Long-term outcome is still unclear. Lifelong antibiotic treatment is mandatory to suppress bacterial load from foreign endovascular bodies.
